# Knowledge, perception and practice of pharmaceutical waste disposal among the public in Lagos State, Nigeria

**DOI:** 10.11604/pamj.2022.42.106.34529

**Published:** 2022-06-08

**Authors:** Halimat Adedeji-Adenola, Afusat Adesina, Margaret Obon, Titilayo Onedo, Gladys Ukamaka Okafor, Michealin Longe, Modupe Oyawole

**Affiliations:** 1 Pharmaceutical Sciences, School of Health Sciences, University of KwaZulu- Natal, Durban, South Africa,; 2 Association of Hospital and Administrative, Pharmacists of Nigeria, Lagos State, Nigeria,; 3 Pharmaceutical Services, Lagos State Primary Healthcare Board, Lagos State, Nigeria,; 4 Department of Pharmaceutical Services, Howard University Global Initiative, Lagos State, Nigeria; 5 Department of Pharmacy, National Orthopaedic Hospital, Igbobi Lagos State, Nigeria,; 6 Pharmacists Council of Nigeria, Lagos Zonal Office, Yaba, Lagos State, Nigeria,; 7 Department of Pharmacy, Onikan Health Centre, Yaba, Lagos State, Nigeria,; 8 Department of Pharmacy, Lagos State University Teaching Hospital, Ikeja, Lagos State, Nigeria

**Keywords:** Disposal practice, knowledge, Lagos State, perception, pharmaceutical wastes

## Abstract

**Introduction:**

increased consumption of pharmaceuticals has been reported to cause a high level of their discharge into the environment, and even small quantities in the environment have the potential to cause harm.

**Methods:**

a descriptive cross-sectional questionnaire-based study was conducted between April and May 2021. The questionnaire was made available online through social media platforms.

**Results:**

a total number of 534 respondents were surveyed. Two hundred and fifty respondents (46.8%) were presently on one or more medications. Many participants have not received advice on pharmaceutical waste from health professionals (413, 78.3%). There is fair knowledge about pharmaceutical waste 234 (43.8%). Many of the respondents think there is a lack of adequate information on what to do with them (500, 93.6%) and there should be a program/ strategy to retrieve all unused, leftover, or expired medicines (475, 88.9%). A lot of respondents throw unused medicines away in household garbage (391, 73.2%)

**Conclusion:**

there is fair knowledge, positive perception, and poor pharmaceutical waste disposal practice. There is a need for the implementation of a “medication take-back program” for appropriate waste disposal practice.

## Introduction

Pharmaceutical waste can be hazardous, non-hazardous, not pharmaceutical active, flammable, irritant, ecotoxic, and harmful. Pharmaceutical wastes include contaminated, unused, unwanted, leftover, expired, prescribed, over-the-counter, drugs, vaccines, and sera that are no longer required and need to be disposed of [1]. Most times, consumers are not able to use all their medications after completing their dosage regimen. Sometimes, medications can be leftover due to non-compliance with the prescribed regimen, adverse drug reactions, or expiration of drugs [2,3]. There are concerns about the fate and effects of these leftovers over known as pharmaceutical wastes on the environment. Studies conducted have shown improper disposal of unused and expired drugs by the public [4,5]. A large portion of the pharmaceuticals detected in waters area from improper disposal of unused drugs by households and medical facilities. They are either flushed down the toilet or thrown into the trash or poured into the zinc [4]. A wide variety of pharmaceutical wastes have been detected in the aquatic environment of various countries, which has shown to have a potential negative impact on humans and animals [4-7]. Studies showed that even small quantities of these pharmaceuticals in the environment have the potential to cause harm [8,9]. Non-steroidal anti-inflammatory drugs (NSAID) such as diclofenac, ibuprofen, and indomethacin have been shown to cause some significant effects in some animals. Diclofenac induced renal failure in vultures after ingestion of cannon of cattle treated with this drug [10]. In an experimental exposure of oriental white-backed vulture to diclofenac residue through feeding with diclofenac treated livestock, renal failure and visceral gout were associated with residues of this anti-inflammatory drug. The study confirms the decline in this creature in Pakistan due to the presence of diclofenac in their feeds [10]. A similar report confirmed a greater than 95% decline in the population of oriental white-backed vulture at Keoladeo National Park in India due to diclofenac in their feeds [11]. Indomethacin at a concentration of 100mg L-1 has been shown to disrupt the oocyte maturation and ovulation process in zebrafish. Also, ibuprofen at a concentration of μg L-1 showed an alteration in the pattern of spawning in Japanese medaka [8]. The presence of Ethinyl estradiol, which is estrogen, is potent in fish. Relevant concentrations have been shown impaired sexual development and feminization in fish [12]. Samples from the Ohio River contained *Escherichia coli* showed resistance to penicillin, tetracycline, and vancomycin [5].

The presence of pharmaceutical chemicals such as hormones, antibiotics, blood lipid regulators, analgesics, anti-inflammatories, beta-blockers, retinoids, tranquilizers, antidepressants, anti-epileptics, and antineoplastic in water bodies have also been detected [9]. Generally, pharmaceutical wastes contaminate the environment including potable water, and pose risk to humans and animals [13,14]. Lagos State, Nigeria has been described as the smallest in terms of land space but the second most populous State, the most economically important State in the country, and it is also ranked among the least livable cities in the world [15,16]. If for instance in Lagos State with a population of over 20 million [17], three-quarters of the residents have unwanted medicines at home and on average each household stores 138.4g as identified by a study carried out in China [18], that will give a total of 2288.4tons of unwanted and unused medicines. Despite the increasing drug development and use of potent pharmaceuticals, some countries do not have official State guidelines for the disposal of these wastes by the community. The Office of National drug control policy (ONDCP) in the United States released official guidance for proper medication safe disposal. This guidance discouraged flushing medication down the toilet and encouraged the community to take advantage of drug take-back programs that allow the public to take unused drugs to a central location for proper disposal [19]. European center for environment and health in France has a practical guide that addresses public health care waste management [6]. New Zealand and Canada have also launched community disposal of unwanted and unused medication programs [20]. The National medicine policy (NMP) in Afghanistan emphasizes the disposal of expired medicines by allocating one percent of the cost of all medicines to be provided in Afghanistan [4]. Consumers lack guidance on the proper disposal of leftover medication. A survey of 301 patients at an outpatient pharmacy in a medical center in Washington DC found that patients stored unused and expired medications at home, twenty (20) percent of respondents have not been advised by health workers about medication disposal, and more than half of patients reported flushing unused medication down the toilet and only 22.9 percent reported returning unused medication to the pharmacy for disposal [2]. A descriptive, cross-sectional survey conducted through face-to-face interviews in Kabul, Afghanistan among public and private employment as well as patients showed that although 98 percent of respondents agreed that improper disposal of unused and expired medicines can affect the environment and health, 77.7% discarded expired medicines in household trash. Many studies conducted on pharmaceutical waste, disposal, and management in Nigeria focus more on health facilities, community pharmacies, and pharmaceutical industries [21-26].

**Aim:** this present study intends to improve the pharmaceutical waste disposal system by assessing the status of pharmaceutical waste disposal among the public in Lagos State.

**Objectives:** i) to assess the level of knowledge and awareness of Lagos residents on pharmaceutical waste disposal; ii) to understand the perception of the public on proper and improper ways of pharmaceutical waste handling; iii) to assess the practice of residents of Lagos on disposal of pharmaceutical waste

## Methods

**Study design and setting:** a descriptive cross-sectional electronic questionnaire-based study was conducted among consented Lagos residents. This study was undertaken in Lagos State, which is an administrative and commercial center of Nigeria, located in the South West of the country. It was conducted between April and May 2021.

**Study population:** the participants were adults irrespective of gender, aged 18 and above with no upper age limit residing in Lagos State. Children and residents below 18 years and any online participants that did not meet the inclusion criteria were excluded by sorting out of the analysis.

**Sampling procedure:** a self-developed anonymous structured questionnaire, containing open and closed-ended questions, was developed. The questionnaire was electronic and made available online through social media platforms such as Facebook, WhatsApp, email, telegram, and Linkedin.

**Sample size determination:** a sample size of 385 participants was computed for a population of 20 million Lagos residents [17] at a 95% confidence interval and 5% margin of error using Rao soft online calculator [27]. The sample size obtained exceeded this estimate. A total of 627 forms were submitted and after sorting out those that stated their residence to be out of Lagos State, 534 completed forms were analyzed in this study.

**Data collection method and instruments:** a survey was carried out using an electronic questionnaire. The tool was adapted from previous similar studies [4,28]. The questionnaire was prepared in the English language. Presented in section 1 was the description of the participants' sociodemographic. knowledge and awareness of pharmaceutical waste were assessed in section 2 while the perception of unused/expired medicines was evaluated in section 3. Section 4 seeks the practice of respondents on pharmaceutical waste disposal. A Pilot study was carried out among 20 participants with a different link demography. The pilot led to changes in the formulation of some questions and options and was not part of the analysis.

**Statistical analysis:** the data were sorted, and all questions were coded and analyzed using statistical package for the social sciences (SPSS) version 24. Descriptive statistics such as frequency and percentage were used to analyze the data. To compare associations between knowledge and sociodemographic, inferential statistics (Chi-square tests) were carried out. A p-value 0.05 was plausible as statistically significant during the data analysis.

**Ethics approval and consent to participate:** ethics approval was obtained from the Lagos State University Research Ethical Review Committee, LREC/06/10/1533. For consent to participate, the purpose and protocols were thoroughly explained on the first page of the electronic questionnaire, and participants that consented had to tick the consent box before proceeding.

## Results

**Sociodemographic information:** a total number of 612 respondents participated in the survey. After sorting out, the number of respondents from Lagos State was 534 making 87.3% of the total respondents. This was used in the analysis of the study. The demographic data showed the number of females was 348, 65.5% and the number of males was 18, 34.8%. The median age was between 18-39 years. Most of the respondents have tertiary education (443, 83%). In the last 3 months before this survey, almost half of the respondents (266, 49.8%) have visited the hospital or pharmacy once or twice, while 94, 17.6% have visited three times or more. Respondents presently on one or more medications were 250, 46.8% while slightly above half of the responses (56.4%) indicated the source of procurement of medicine to be from the pharmacy with prescriptions (Table 1).

**Table 1:** sociodemographic of respondents (N=534)

Variables and categories		Number of responses (%)
**Gender**	Female	348 (65.2)
	Male	186 (34.8)
**Ager range**	18-39	338 (63.3)
	40-49	104 (19.5)
	50-59	63 (11.8)
	>59	29 (5.4)
**Education**	Primary	5 (0.9)
	Secondary	47 (8.8)
	Tertiary	443 (83.0)
	No formal education	39 (7.3)
**Number of medicines taken at present**	0	284 (52.2)
	1-5	239 (44.8)
	6-10	10 (1.9)
	>10	1 (0.2)
**Number of hospital or pharmacy visits in the last 3 months**	0	174 (32.6)
	1-2 times	266 (49.8)
	3- 4 times	66 (12.4)
	>4 times	28 (5.2)
**Number of unused/expired medicine presently at home**	0	251 (47.0)
	1-5	233 (43.6)
	6-10	37 (6.9)
	11-25	9 (1.7)
	>25	4 (0.7)
*Source of medicine procurement	From the pharmacy with prescription	443 (56.4)
	Over the counter without a prescription	157 (20.0)
	Receive from friends/colleagues	71 (9.1)
	Purchase at any drug store	34 (4.3)
	Purchase based on advice from friends/ relatives	60 (7.7)
	Others	20 (2.5)
*Class of medicine frequently used	Multivitamin/supplements	250 (31.6)
	Medicine for pain	203 (25.6)
	Antihypertensives	58 (7.3)
	Antibiotics	206 (26.0)
	Others	75 (9.5)

**Analysis of knowledge of respondents on pharmaceutical wastes:** more than half of respondents have one or more unused/expired drugs presently at home. Two hundred and thirty-three respondents (233) which is 43.6% have between 1-5 unused/expired medications at home while 50, 9.4% have more than 5 (Table 1). Respondents that are females (273, 51.1%) check the expiry dates of products before buying than males (112, 20.9%). Almost half of the respondents know what pharmaceutical wastes are (266, 49.6%). The majority of participants have not received advice on pharmaceutical waste from health professionals (413, 78.3%), have not read instructions on the disposal of unused medicines (378, 70.8%) and many do not know what to do with unused/ expired medicines (309, 57.9%). The average positive response for knowledge was 234 (43.8%) (Table 2).

**Table 2:** association between knowledge of pharmaceutical wastes and demographic characteristics for positive responses N=534

Statements		Gender		Age			Education			
	Total yes(%)	Male	Female	18-39	40-59	>59	Primary	Secondary tertiary		No formal education
Check expiry dates before buying drugs	384 (71.9%)	112 (20.9%)	273 (51,1%)	224 (41.9%)	13 (2.4%)	25 (4.7%)	1 (0.2%)	29 (5.4%)	323 (60.5%)	32 (6.0%)
		p=0.000*		p=0.006*			p=0.77			
Receive advise from health workers on disposal of pharmaceutical wastes	116 (21.7%)	43 (8.0%)	73 (13.7%)	5 (0.9%)	74 (13.8%)	37 (6.9)	0 (0.0%)	9 (1.7%)	96 (18.0%)	11 (2.0%)
		p=0.568		p=0.947			p=0.467			
Know what pharmaceutical wastes are	266 (49.8%)	81 (15.2)	185 (34.6%)	167 (31.3%)	83 (15.5%)	16 (2.0%)	0 (0.0%)	13(2.4%)	232(43.4%)	21 (3.9%)
		p=0.034*		p=0.939			p=0.001*			
Read instructions on pharmaceutical wastes	156 (29.2%)	52 (9.7%)	104 (19.5%)	91 (17.0%)	55 (10.3%)	10 (1.9%)	0(0.0%)	12 (2.2%)	133 (24.9%)	11 (2.1%)
		p=0.641		p=0.300			p=0.476			
Know what to do with unused/expired drugs	225 (42.1%)	74 (13.8%)	151 (28.3%)	123(23.0%)	85 (15.9%)	17 (3.2%)	1 (0.2%)	16 (3.0%)	187 (35.0%)	21 (3.9%)
		p=0.421		p=0.004*			p=0.216			
Know unused/ expired drugs affects environment	256 (47.9%)	90 (16.8%)	166 (31.1%)	174 (32.6%)	73 (13.7%)	9 (1.7%)	1(0.2%)	19 (3.6%)	218 (40.8%)	18 (3.4%)
		p=0.880		p=0.77			p=0.397			

Average yes 234 (43.8%)

**Analysis of perception concerning unused or expired medication:** most of the respondents 458 (85.8%) feel they present potential risks at home and children are more vulnerable to risks associated with them (501, 93.8%). The majority of respondents think there is a lack of adequate information on what to do with them (500, 93.6%) and there should be a program/strategy to retrieve all unused, leftover, or expired medicines (475, 88.9%) (Table 3).

**Table 3:** respondents' perception of unused/expired medications

Statements	Strongly agree/agree	Neither agrees/disagree	Strongly disagree/disagree
	n (%)	n (%)	n (%)
Unused and expired medicines present potential risks at home	458 (85.8)	64 (12)	12 (2.3)
Children are more vulnerable to risks associated with unused and expired household medicines	501 (93.8)	23 (4.3)	10 (1.9)
There is a lack of adequate information on what to do with unused and expired household medicines	500 (93.6)	19 (3.6)	15 (2.8)
Healthcare professionals advise on what to do with unused and expired household medicines	125 (23.4)	107 (20.0)	302 (56.6)
There should be a program/ strategy to retrieve all unused, leftover, or expired medicines	475 (88.9)	43 (8.1)	16 (3.0)

N: sample size; n: number of responses

**Figure 1:**
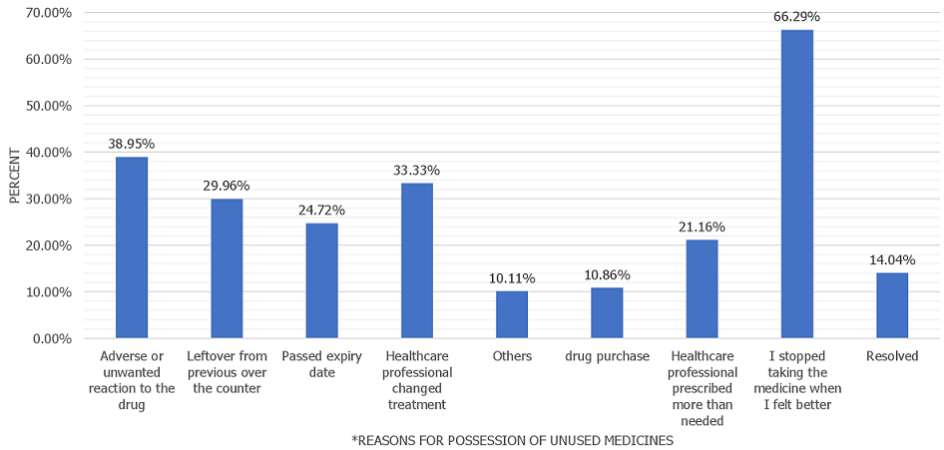
reasons for possession of unused medicine at home

**Analysis of reasons for keeping unused medicines:** major reasons respondents kept unused medicine at home were because they stopped taking medicine when they got better and because the ailment resolved (429, 80.3%), due to adverse drug reactions (208, 39%), because health professionals changed their treatment (178, 33.3%) and because health professionals prescribed more than what was needed (113, 21.2%) (Figure 1).

**Pharmaceutical waste disposal practice among respondents:** a lot of respondents throw unused medicines away in household garbage (391, 73.2%), keep them at home until expired (164, 30.7%) and flushed them into the toilet/sink (103, 19.3%) (Table 4). Before discarding expired/ unused medicines, some don´t know what to do (118, 22.1%), many respondents discard as it is (333, 62.4%), crash before discarding (109, 17.7%) and dilute them with water before discarding (49, 8.6%) (Table 4).

**Table 4:** respondents' practices concerning unused and leftover medicines

Questions		N (%)
Did any quantity of purchased medicine remain unused at home?	Yes	283 (53.0)
	No	251 (47.0)
What do you do with the unused medicines?	Burn	22 (4.1)
	Return to pharmacy	18 (3.4)
	Donate to hospital	12 (2.2)
	Throw away in household garbage	391 (73.2)
	Flush unused medications in the toilet/sink	103 (19.3)
	Give to friends or relatives	42 (7.9)
	Keep at home until expired	164 (30.7)
	Other	24 (4.5)
What do you do before discarding expired or unused medicines?	As it is	333 (62.4)
	I don't know what to do	118 (22.1)
	Other	40 (7.5)
	Crashed before discarding	91 (17.0)
	Diluted with water	46 (8.6)

## Discussion

Increased consumption of pharmaceuticals has been reported to cause a high level of their discharge into the environment and studies have shown that even small quantities of these pharmaceuticals in the environment have the potential to cause harm [5,9,14]. This research was inspired due to increased requests from healthcare users seeking how to manage their expired products. This study was carried out to seek probable policy solutions that can improve the pharmaceutical waste disposal system, by first assessing the status of pharmaceutical waste disposal among the residents of Lagos State. In this study, findings showed that 71.9% check the expiry dates of medications before buying. Bashar *et al*., 2017 reported in Kabul, Afghanistan that 97% of respondents check the expiry dates of medicine before procurement [4], Insani *et al*., 2020 revealed that 72.8% check the drug expiry date in a study carried out in Indonesia [29]. However, a contrast study carried out in India showed participants are not aware of medication expiry dates [30]. It is important to be aware of expiry dates, shelf life and expired products to prevent drug poisoning, adverse drug reactions and to ensure potent medicines are used [31,32]. The massive raids of pharmacies and shops by the Nigerian customs service (NCS) and the National Agency for Food and Drug Administration and Control (NAFDAC) showed there have been increased negligent sales, deliberate relabelling and revalidating dates of expired foods and drugs. The high positive response of participants to checking expiry dates before the purchase of medicines could be due to various news media stories, experience, and advocacy on the need to always check drug´s expiry dates [33-35]. Findings from this study showed that 53% of respondents presently have unused/expired drugs at home. In a similar study carried out to assess the knowledge, attitude and practice of residents of Maiduguri in North-eastern Nigeria, 35% of respondents stored unused medications at home for future use or till expiry [36]. In a survey carried out at the outpatient pharmacy of a medical center in Fort Lewis WA, findings showed that half of the respondents keep unused drugs at home [2]. In Badung, Indonesia, data collected through interviews of 497 respondents also showed that 95.5% stored unused/ expired medicines at home [29]. According to a study in Ireland, 23% of medicines kept were invalid [37]. Keeping unused medicine at home has been identified as a socio-economic problem that is associated with incomplete recovery, self-medication, intentional or accidental drug poisoning, and environmental pollution [38-41].

This study showed that 80.3% of respondents kept unused/leftover medicines at home because they felt better, or their ailment was resolved while 39.0% kept unwanted drugs because of adverse drug reactions to the drugs when they took them. An easier way to report adverse drug reactions is by encouraging users to report directly to the pharmacovigilance authorities [42]. This will ensure prompt feedback, when necessary, the direct reporting method can also be a medium to further educate them on what to do [43] and can also serve as a means of building confidence and trust in the medicine. In a previous similar study, another credible reason for keeping leftover/ unwanted drugs in Nigeria was to save cost because medicines are purchased out-of-pocket by most Nigerians due to low health insurance coverage medicines are also kept at home due to common self-medication practice [36]. A survey in New Zealand showed main reason for people (67.9%) to have leftover medication was due to improve their medical condition or resolved it [44]. Importantly, keeping unused drugs at home could suggest inadequate counselling of patients on the importance of drug completion. It also indicates medication non-adherence, especially in people that keep medicines because they felt better. Halting medication use due to adverse drug reactions is also an issue to emphasize. Non-compliance with prescribed dosage and duration does not only lead to the accumulation of unused/expired drugs that can pose risks to people and the environment, but it can also result in treatment failure, re-infection, additional health expenditure and poor health outcomes [45,46]. Also, in this study, 21.2% of participants keep unused drugs at home because health professionals prescribed an excess of the medicine. The economic cost of medication wastage in Saudi Arabia had been estimated to be $150 million annually [47]. Appropriate and rational prescribing and dispensing will minimize medication wastage and prevent or reduce keeping unused drugs at home [48]. Leveraging on individual or unit dose dispensing which ensures patient-specific dosages of drugs are dispensed can prevent or minimize having unused medicines at home. Unit dose dispensing should not only be for inpatients, but should be extended to all healthcare users in public and private health facilities. Findings from this study showed most respondents perceived that the presence of unused and expired medicines presents potential risks to which children are more vulnerable. Similar findings reported by a study in North-Eastern Nigeria also showed the majority of respondents know there are dangers and health risks associated with leftover medicines [36].

Pregnant women and children have been identified as vulnerable to pharmaceutical wastes [49]. Despite the perception of associated risks posed by pharmaceutical wastes (85.3%), this study showed poor practice concerning pharmaceutical waste disposal among the public surveyed in Lagos State. There is varied disposal practice among respondents. On what they do with leftover/unused medicines, the majority (73.2%) of respondents reported throwing them away in the garbage, keep at home until expired (30.7%), flush into the toilet (19.3%) and so on. This is like practice in Kabul, Afghanistan, Bugan City of Korea, Selangor Malaysia and the North-eastern part of Nigeria [4,36,50,51]. Concerning what respondents do before discarding pharmaceutical wastes, many (62.4%) dispose of them as it is, little crush them before discarding (17.0%), dilute them with water (8.6%), while some do not know what to do (22.1%). There is a lack of adequate information for people on what to do with unused medicines. Respondents alleged healthcare professionals of not giving advice/information on this issue (56.6%). Healthcare professionals are best suited to guide and educate healthcare users on medication disposal. Pharmacists being drug experts have been identified as professionals that can tackle pharmaceutical waste issues by recommending appropriate disposal processes to patients and caregivers [52,53]. Unavailability of established disposal practice has been identified as a probable reason for inappropriate disposal, and it has been suggested that mixing medicine with unappealing substances before throwing in household garbage may be a fair alternative to curb another person picking them up for use [36]. Medicine/ Drug take-back programs have been identified as the best method of disposing of unused/unwanted/leftover or expired medicines [4,54]. In the United States of America (USA), the Drug Enforcement Administration (DEA) ensure temporary collection sites are set up in the community while for permanent collection sites healthcare users dispose of unused medicines to DEA registered collectors [55]. A study carried out in New Zealand showed, depending on formulation type, 13- 24% of medications are returned to a pharmacy [44]. Australia has a medication take-back program called return unwanted medicines. It was reported that the campaign successfully collected 700 tons of unwanted medicines in 2015-2016 [56]. In Saudi Arabia, some pharmacies collect medications from patients for proper disposal, the pharmaceutical care services department at King Abdul-Aziz Medical City in Jeddah also has a policy that patients return unused medicines [54]. In Nigeria, the National Environmental Standards and Regulations Enforcement Agency (NESREA) regulates waste management, including pharmaceutical wastes. In a paper presentation in 2017 which focused on pharmaceutical waste, the Agency stipulates that persons and facilities that generate medical wastes are responsible for the collection and disposal of such waste [57]. It appears that Nigerian residents take responsibility for their pharmaceutical waste disposal. There should be public education on the appropriate disposal of medicines. There is a need for the establishment of a national policy on pharmaceutical waste disposal. There should be an official and mandatory protocol that will guide the public on medicine disposal. Nigeria can adopt a well-planned program such as “take-back medicines” in the communities, where designated outlets (such as community pharmacies and primary healthcare centers) can be used as collection points of unused/ expired/ leftover medicines. This will ensure pharmaceutical wastes are collected for appropriate disposal exercises.

**Study limitation:** the initial proposal was to administer the questionnaire in healthcare facilities in Lagos State to have fair representation across different demography, but it was not possible due to the present COVID-19 pandemic. The electronic survey had eighty-three (83) per cent of respondents with tertiary education. This might imply unfair representation in the findings. The poor disposal practice response obtained despite the level of education however may imply there might not be a better response.

**Funding:** Association of Hospital and Administrative Pharmacists (AHAPN), Lagos State branch funded this research.

## Conclusion

Respondents have inadequate knowledge of pharmaceutical wastes, positive perception of unused/expired medications and poor pharmaceutical waste disposal practices. The results from this study showed the need for government and policymakers to intensify efforts on appropriate waste disposal practices in Lagos State. There should be continuous education and awareness program for health workers and the public on the pharmaceutical waste system.

### What is known about this topic

- Previous studies have shown improper disposal of leftover, unused and expired medicines by the consumers;
- The presence of these products is hazardous and harmful to the environment;
- Many studies conducted on pharmaceutical waste, disposal and management in Nigeria focus more on health facilities, community pharmacies and pharmaceutical industries.

### What this study adds

- There is inadequate knowledge and poor pharmaceutical waste disposal practices by residents of Lagos State, Nigeria; public education on the appropriate disposal of medicines will enhance the safety and protection of people and the environment;
- This study provides a reference for governments and policymakers to establish and implement policies and systems that will encourage the public to dispose of leftover/unwanted/unused or expired medicines safely; proper pharmaceutical waste disposal and management can help the Government to also estimate the cost of these products; this will stimulate an active move to reduce pharmaceutical wastes to the barest minimum;
- Stakeholders can adopt “take back medicines”, a program that provides designated places in the community for pharmaceutical waste collection and appropriate disposal exercises.
